# A clinical prediction model for 90-day functional dependence after minor stroke/TIA with exploratory pharmacogenetic associations: a post-hoc analysis of the INSPIRES trial

**DOI:** 10.3389/fnagi.2026.1922100

**Published:** 2026-10-14

**Authors:** Yaping Wang, Baizhu Li, Hongyi Yan, Lingling Jiang, Yuesong Pan, Yilong Wang

**Affiliations:** 1 Beijing Tiantan Hospital, Capital Medical University, Beijing, China; 2 Department of Neurology, Beijing Tiantan Hospital, Capital Medical University, Beijing, China; 3 China National Clinical Research Center for Neurological Diseases, Beijing, China; 4 Chinese Institute for Brain Research, Beijing, China; 5 National Center for Neurological Disorders, Beijing, China; 6 Advanced Innovation Center for Human Brain Protection, Capital Medical University, Beijing, China; 7 Beijing Laboratory of Oral Health, Capital Medical University, Beijing, China; 8 Laboratory for Clinical Medicine, Capital Medical University, Beijing, China

**Keywords:** dual antiplatelet therapy (DAPT), functional outcome, minor stroke, pharmacogenetics, prediction model, risk stratification, transient ischemic attack (TIA)

## Abstract

**Background and purpose:**

Dual antiplatelet therapy (DAPT) reduces stroke recurrence after minor stroke/TIA, yet a clinically meaningful proportion of patients remain functionally dependent at 90 days. We developed an interpretable clinical prediction model to define a high-risk subgroup, and separately explored pharmacogenetic associations within this subgroup to generate hypotheses for precision secondary prevention.

**Methods:**

In this post-hoc analysis of the INSPIRES trial (4,439 patients), LASSO logistic regression selected predictors for 90-day functional dependence (mRS > 2). Model performance was assessed by discrimination, calibration, and decision curve analysis. In a genotyped subcohort, CYP1A2 rs762551 and POR rs1057868 were evaluated for outcome association and enrichment in the high-risk subgroup.

**Results:**

Logistic regression achieved the highest AUC point estimate (AUC = 0.727) among the four models evaluated (AUC range: 0.495–0.698; 95% CIs overlapped across models); it was selected as the final model for its interpretability and calibration, rather than for statistical superiority. Key predictors were admission NIHSS, age >65 years, and diabetes. The highest-risk quartile (25% of patients) carried a disproportionate outcome burden, with no effect modification by antiplatelet strategy (*p* = 0.793). CYP1A2 CC (23.65% vs. 16.28%; Pearson *χ*^2^*p* = 0.0031) and POR TC (54.58% vs. 46.22%; Pearson *χ*^2^*p* = 0.0105) genotypes were enriched in functionally dependent patients; CYP1A2 showed no significant allele-dosage trend (trend *p* = 0.0821), whereas POR demonstrated a significant allele-dosage trend (*p* = 0.0032).

**Conclusion:**

This clinical prediction model identified a high-risk subgroup accounting for most poor outcomes after minor stroke/TIA. Exploratory analyses identified CYP1A2 and POR variants enriched in this subgroup, generating pharmacogenetic hypotheses for future investigation.

## 1 Introduction

The early phase after ischemic stroke or high-risk transient ischemic attack (TIA) is a critical window for intensifying secondary prevention to reduce early recurrence and improve longer-term outcomes (Mendelson and Prabhakaran, 2021; Zhou et al., 2025; Lee Y. et al., 2024; Lun et al., 2022). Short-term dual antiplatelet therapy (DAPT) with aspirin plus clopidogrel is guideline-recommended and reduces the risk of recurrent ischemic events in selected high-risk patients (Zheng et al., 2021; Hobeanu et al., 2022; Chen et al., 2023; Eastin, 2023; Valgimigli et al., 2022). In the INSPIRES trial, 186 patients (4.19%) remained functionally dependent (mRS > 2) at 90 days despite receiving standard DAPT. Across prior studies, the proportion of minor stroke/TIA patients with unfavorable 90-day functional outcomes ranges from approximately 3 to 8% (Chen et al., 2023; Gao et al., 2023). This pronounced inter-individual variation in residual functional risk suggests that a “one-size-fits-all” approach may overlook baseline clinical and biological factors that shape prognosis.

The INSPIRES trial (Improving Stroke Prevention With Intensive Statins and Dual Antiplatelet Therapy, NCT03635749) is a multicenter, randomized, double-blind, 2 × 2 factorial clinical trial conducted at 222 sites across China, enrolling 6,100 patients with acute mild-to-moderate ischemic stroke or high-risk TIA within 72 h of onset. The primary research questions were: (1) whether clopidogrel plus aspirin (DAPT) is superior to aspirin alone, and (2) whether immediate intensive statin therapy (initiated within 24 h of onset) is non-inferior to delayed intensive statin therapy (initiated 72 h after admission). The primary efficacy endpoint was new stroke events at 90 days. The detailed trial design has been described in previously published protocols and core trial reports (Elias et al., 2022). The present study uses data from the full INSPIRES dataset for a pre-specified post-hoc analysis.

We hypothesize that poor functional outcome risk is systematically driven by a baseline multidimensional risk profile. This profile may reflect cerebral reserve (e.g., neuroimaging markers such as white matter hyperintensity and cerebral atrophy, and acute deficit severity measured by the NIHSS) (Voorter et al., 2025; Bagnato et al., 2024; Baumeister et al., 2024) as well as factors influencing recovery potential (e.g., age, hypertension, and diabetes) (Kedhi et al., 2021; Cheng et al., 2023). Prediction models that identify a clinically meaningful high-risk subgroup for functional dependence could enable individualized management by prioritizing intensified multidisciplinary prevention and follow-up in those with the greatest residual risk, including patients who may remain vulnerable despite standard antiplatelet strategies (Park et al., 2020; de Jong et al., 2021; Gao et al., 2024).

To address this need, we used data from the prospective, multicenter INSPIRES trial (Elias et al., 2022; Butala et al., 2022) to develop a “clinical prediction–pharmacogenetic association” framework. First, we integrated multimodal baseline features to develop an interpretable model for individualized prediction of 90-day functional dependence and to define a high-risk subgroup. Second, we explored whether genetic variation across distinct biological pathways is associated with poor functional outcomes and enrichment within the model-defined high-risk subgroup. Specifically, we examined: (1) a variant in a phase I metabolic enzyme pathway, CYP1A2 (rs762551), which is a minor contributor to clopidogrel oxidative metabolism; its potential role in functional recovery may also involve broader metabolic or regulatory pathways, although this remains speculative and may contribute to inter-individual variability (Khasawneh et al., 2024; Subash et al., 2025); and (2) POR (rs1057868) encodes the unique electron donor to CYP enzymes and may indirectly modulate neurorepair and functional recovery by affecting drug metabolism and endogenous signaling pathways (Guerri et al., 2023).

Accordingly, this study adopted a two-stage analytic strategy: the first stage involved developing a clinical prediction model using multimodal baseline features to identify a high-risk subgroup for functional dependence; the second stage involved exploring, in an independent genotyped subcohort, whether specific pharmacogenetic variants are enriched among patients with unfavorable outcomes. By separating clinical prediction from genetic exploration, we aimed to first define the high-risk phenotype clinically, then generate biologically informed hypotheses about potential genetic contributors to residual risk, thereby supporting future prospective studies of tailored secondary prevention strategies.

## 2 Methods

### 2.1 Study design and participants

This study was a prespecified secondary analysis of the INSPIRES trial, a multicenter, randomized, double-blind, 2 × 2 factorial clinical trial conducted at 222 sites in China between September 17, 2018, and October 15, 2022. The trial protocol was approved by the Ethics Committee of Beijing Tiantan Hospital and the institutional review boards of participating centers (Approval No. KY2017-065-02).

The INSPIRES trial adopted a 2 × 2 factorial randomized design with two independent intervention factors. The first factor was antiplatelet strategy, which randomly assigned eligible patients to either dual antiplatelet therapy (clopidogrel plus aspirin) or single antiplatelet therapy (aspirin alone). The second factor was statin intervention timing, which randomly assigned patients to either immediate intensive statin therapy initiated within 24 h after symptom onset, or delayed intensive statin therapy initiated 72 h after admission. The key inclusion and exclusion criteria of the INSPIRES trial have been described in detail in previously published core trial reports. The primary analysis focus of the current post-hoc study was the association between baseline risk stratification and 90-day functional outcome, as well as the potential effect modification of antiplatelet strategy across different risk strata, while the statin treatment allocation was adjusted as a predefined covariate in all multivariable models to control for its potential confounding effect. Both the core trial report covering antiplatelet strategy efficacy and the published report focusing on statin intervention outcomes have been cited in the reference list of this manuscript to comprehensively document the complete design of the source trial.

For the prediction analysis, participants were included if they had complete 90-day follow-up and complete baseline data for prespecified key clinical variables and neuroimaging features. The primary outcome was 90-day functional dependence, defined as modified Rankin Scale (mRS) score >2, assessed according to the trial follow-up procedures.

Genetic association analyses were conducted in a separate full screening cohort with available genotype data for prespecified loci and available 90-day functional outcome data. This full screening cohort comprised all INSPIRES patients who successfully underwent genotyping for the targeted loci (*n* ≈ 5,856 for CYP1A2 and *n* ≈ 5,844 for POR), representing the primary genetic analysis cohort for the systematic SNP screening. From this full screening cohort, a validation subcohort (*n* = 2,348) was randomly selected for baseline characteristic comparisons and sensitivity analyses. Of note, the genetic subcohort was not nested within the prediction cohort, as it included patients who had genotype data but may have lacked complete imaging or other clinical variables required for the prediction model. The genetic analyses were therefore performed as an independent exploratory investigation, rather than as an integrated component of the prediction model development.

### 2.2 Data collection

Baseline data included demographics, vascular risk factors and medical history, clinical severity measures (admission NIHSS and prestroke mRS), socioeconomic variables (education level and insurance status), neuroimaging markers (white matter hyperintensity burden, cerebral atrophy, lacunes, and cerebral microbleeds), and blood samples for genotyping.

### 2.3 Clinical prediction model

#### 2.3.1 Candidate predictors and preprocessing

Candidate predictors were prespecified based on clinical relevance and availability at baseline. Continuous predictors were retained on their original scale where appropriate, and categorical predictors were dummy encoded. (If applicable) Analyses were conducted using complete-case data.

#### 2.3.2 Predictor selection using LASSO logistic regression

To reduce overfitting and perform data-driven variable selection, we applied least absolute shrinkage and selection operator (LASSO) logistic regression in the training dataset. The optimal penalty parameter (*λ*) was selected using k-fold cross-validation (*k* = 5) based on the minimum cross-validated deviance (*λ*_min). Predictors with non-zero coefficients under the selected *λ* were retained as the final feature set.

#### 2.3.3 Model development and internal validation

##### 2.3.3.1 Handling of class imbalance

Given the low incidence of the primary outcome (4.19%), we implemented multiple strategies to address class imbalance. First, we applied outcome-stratified splitting to ensure that the proportion of functional dependence events was consistent between the training (70%) and test (30%) sets. Second, for all algorithms, we incorporated class weights (class_weight = “balanced”) to assign higher penalty to misclassification of the minority class. Third, as a sensitivity analysis, we applied the Synthetic Minority Over-sampling Technique (SMOTE) to the training set to assess the robustness of our findings. Results from the SMOTE analysis were consistent with the main analysis (data not shown), suggesting that our conclusions were not sensitive to the specific imbalance handling strategy.

The dataset was randomly split into training and test sets (70:30) using outcome-stratified sampling. Using the LASSO-selected predictor set, we developed an interpretable multivariable logistic regression model to predict 90-day functional dependence. For benchmarking, additional machine learning models (random forest, support vector machine, and XGBoost) were trained in the training set, with hyperparameters tuned using 5-fold cross-validation with minimization of cross-validated log-loss as the optimization objective. AUC was used only as a discrimination metric for final model performance reporting to facilitate clinical interpretation and comparison with prior studies. Detailed preprocessing and hyperparameter tuning procedures were as follows: (a) Feature scaling for SVM: all continuous predictors (e.g., age, NIHSS score) were standardized to mean 0 and SD 1 using the scale() function prior to SVM training, which is a standard preprocessing step for SVM with the radial basis function (RBF) kernel. (b) SVM hyperparameters: the RBF kernel was used, with the cost parameter C (range: 2^−5^ to 2^15^) and kernel parameter *γ* (range: 2^−15^ to 2^3^) searched via 5-fold cross-validation on the training set to maximize cross-validated AUC. The final selected parameters were *C* = 4 and *γ* = 0.125. (c) Random forest hyperparameters: the following parameters were searched via 5-fold cross-validation: mtry ({2, 4, 6, 8, 10}), ntree ({100, 200, 500}), and nodesize ({5, 10, 20}). The final selected parameters were mtry = 6, ntree = 500, and nodesize = 10. (d) XGBoost hyperparameters: the following parameters were searched via 5-fold cross-validation: learning rate (eta, {0.01, 0.05, 0.1}), maximum tree depth (max_depth, {3, 5, 7}), subsample ratio ({0.6, 0.8, 1.0}), column sampling ratio (colsample_bytree, {0.6, 0.8, 1.0}), and L2 regularization parameter (lambda, {0, 1, 5}). The final selected parameters were eta = 0.05, max_depth = 5, subsample = 0.8, colsample_bytree = 0.8, and lambda = 1. All hyperparameters were optimized using cross-validated AUC on the training set. No formal pairwise statistical comparisons were performed between models, as the goal was not to identify a single “best” algorithm but rather to select a parsimonious, interpretable model for clinical application.

Model discrimination was assessed using AUC with 95% CIs. Calibration was evaluated using calibration plots comparing predicted versus observed risk, with calibration slope and calibration-in-the-large quantified to supplement visual assessment. Clinical utility was assessed using decision curve analysis across a range of threshold probabilities. All performance evaluations were conducted in the held-out test set.

#### 2.3.4 Model interpretation

To enhance interpretability of the final logistic regression model, we primarily relied on odds ratios and their 95% confidence intervals, as logistic regression parameters directly provide interpretable effect estimates. In addition, Shapley Additive Explanations (SHAP) were applied as a supplementary visualization tool to illustrate individual-level predictor contributions, with results provided in Supplementary Figure S2. The SHAP analysis serves only to improve the interpretability of the multivariate model and does not substitute for standard statistical inference based on formally calculated regression coefficients.

### 2.4 Baseline risk stratification and treatment effect modification

A model-derived baseline risk score was used to stratify participants into prespecified risk categories (e.g., quartiles). Multivariable logistic regression was used to assess the association between baseline risk strata and outcome, and to test for potential effect modification by antiplatelet strategy (DAPT vs. single antiplatelet therapy) using an interaction term (risk strata × treatment), adjusting for prespecified covariates including age, hypertension, diabetes, and baseline NIHSS.

### 2.5 Pharmacogenetic association analyses

A systematic candidate gene screening was performed in patients with available genotyping data from the INSPIRES trial. The gene panel was selected based on prior literature reporting associations with clopidogrel pharmacokinetics, platelet function, or stroke recovery, encompassing metabolic enzymes (CYP450 family), drug transporters (ABCB1, among others), and coagulation pathway genes. A total of 107 single nucleotide polymorphisms (SNPs) were included, covering loci such as CYP2C19, CYP3A4, CYP3A5, CYP1A2, POR, ABCB1, PON1, and ITGB3. All SNPs were genotyped using TaqMan probe assays and quality-controlled via Hardy–Weinberg equilibrium testing (*p* > 0.05). Gene-outcome association analyses were performed using an additive genetic model, with Bonferroni correction (*α* = 0.05/107 ≈ 0.00047) applied to determine the significance threshold. Among the 107 SNPs, only CYP1A2 rs762551 (*p* = 0.0031, adjusted *p* = 0.33) and POR rs1057868 (*p* = 0.0105, adjusted *p* = 1.0) showed potential association signals, warranting further investigation.

CYP1A2 (rs762551) and POR (rs1057868) were evaluated for associations with 90-day functional outcome and for enrichment in the model-defined high-risk subgroup. These two loci were selected for focused reporting because they yielded the smallest *p*-values among all screened SNPs and demonstrated biological plausibility related to clopidogrel metabolism (CYP1A2) and cytochrome P450 electron donation (POR). Genotype distributions between outcome groups were first compared using *χ*^2^-based tests (Pearson *χ*^2^ and likelihood-ratio *χ*^2^) and Mantel–Haenszel trend testing. To account for potential confounding, we then performed multivariable logistic regression adjusting for key clinical covariates identified in the prediction model, including age, baseline NIHSS score, and diabetes. Results are reported as adjusted odds ratios with 95% confidence intervals. We applied Bonferroni correction for multiple comparisons, with a two-sided *p* < 0.025 considered statistically significant. Biological interpretation was informed by prior literature, and genetic findings were considered hypothesis-generating. Standard genetic quality control procedures were implemented in this analysis. First, Hardy–Weinberg Equilibrium (HWE) testing was performed to assess genotype distribution conformity in the total study population, with *p* > 0.05 defined as consistent with population genetic equilibrium. Second, given that only 2 predefined candidate loci were analyzed, Bonferroni correction was applied for multiple testing, setting the adjusted statistical significance threshold at *α* = 0.025 (0.05 divided by the number of tested loci). Third, the observed allele frequencies in our cohort were compared with publicly available reference data from the East Asian population subset of the gnomAD database to confirm the consistency of genotype distribution with local population characteristics.

### 2.6 Statistical analysis

All analyses were performed using R (version 4.1.0). LASSO regression was implemented using the glmnet package (version 4.1). Random forest was implemented using the randomForest package (version 4.7), XGBoost using the xgboost package (version 1.6), and support vector machine using the e1071 package (version 1.7-3). SHAP visualizations were generated using the shapviz package (version 0.9). Logistic regression results are reported as odds ratios (ORs) with 95% confidence intervals (CIs). Model performance is reported using AUC with 95% CIs, calibration, and decision curve analysis. A two-sided *p* < 0.05 was considered statistically significant. To evaluate the impact of missing data, we performed a sensitivity analysis using multiple imputation (MICE method, 10 imputations) for covariates with missing values (WMH grade, cerebral atrophy, lacune status, and cerebral microbleeds). The imputation model included all outcome variables and candidate predictors. Results from the multiply imputed datasets were compared with the complete-case analysis. The analysis code and data are available from the corresponding author upon reasonable request.

## 3 Results

### 3.1 Study population and baseline characteristics

Baseline characteristics were compared between patients with functional independence and functional dependence at 90 days. Patients with functional dependence differed in several clinical and imaging features, including older age, higher prevalence of hypertension and diabetes, differences in education level distribution, greater white matter hyperintensity (WMH) burden, and differences in cerebral atrophy and lacunar status, as well as higher admission NIHSS and prestroke mRS scores (Table 1). The patient screening flowchart is shown in Figure 1. Of the 6,100 patients enrolled in the INSPIRES trial, 4,439 were included in the final prediction cohort after excluding those without complete 90-day follow-up and those with missing key baseline variables, among whom 186 (4.19%) had 90-day functional dependence (mRS > 2). The test set comprised 1,399 patients (30% of the prediction cohort), including 74 functional dependence events (5.29%); the slightly higher event rate in the test set compared to the full cohort (4.19%) reflects normal random variation in stratified sampling. Missing data for each baseline variable are summarized in Supplementary Table S1; the proportions of missing values were generally low (most variables <3%, with imaging variables <5%).

**Table 1:** A study on predicting functional dependence in 90-day period for patients with ischemic stroke: baseline characteristics of the study population (stratified by 90-day functional outcome).

Variable	Functional independence group (*N* = 4,253)	Functional dependence group (*N* = 186)	Statistic	*p*-value
Demographics				
Age (years), median (IQR)	65.00 (57.00–71.00)	69.00 (63.00–74.00)	*Z* = 5.35	<0.001
Age >65 years, *n* (%)	2,108 (49.57%)	122 (65.59%)	*χ*^2^ = 17.6	<0.001
Gender (male), *n* (%)	2,714 (63.81%)	106 (56.99%)	*χ*^2^ = 3.60	0.0584
Medical history, *n* (%)				
Hypertension (H_HYPT01)	2,862 (67.29%)	148 (79.57%)	*χ*^2^ = 12.1	0.0005
Diabetes (H_DIAB01)	1,139 (26.78%)	71 (38.17%)	*χ*^2^ = 11.6	0.0006
Baseline clinical				
Admission NIHSS score (A_NIHSS), median (IQR)	2.00 (0.00, 3.00)	3.50 (2.00–5.00)	*Z* = 10.9	<0.001
Pre-stroke mRS score (A_MRS), median (IQR)	0.24 ± 0.51	0.38 ± 0.60	*Z* = 3.59	0.00021
Education level (B_EDUCATION), *n* (%)			*χ*^2^ = 13.4	0.0098
Level 1 (illiterate)	146 (3.43%)	3 (1.61%)		
Level 2 (primary school)	487 (11.45%)	18 (9.68%)		
Level 3 (junior high school)	1,448 (34.05%)	49 (26.34%)		
Level 4 (senior high school)	1,547 (36.37%)	74 (39.78%)		
Level 5 (university or above)	625 (14.70%)	42 (22.58%)		
Insurance type (B_MEDICARE), *n* (%)			*χ*^2^ = 2.58	0.7723
Level 1	709 (16.67%)	25 (13.51%)		
Level 2	1,048 (24.64%)	48 (25.95%)		
Level 3	2,352 (55.30%)	108 (58.38%)		
Level 4	5 (0.12%)	0 (0.00%)		
Level 5	7 (0.16%)	0 (0.00%)		
Level 6	132 (3.10%)	4 (2.16%)		
Imaging features, *n* (%)				
White matter hyperintensity (Fazekas grade 0: absent)‌	21 (0.49%)	0 (0.00%)	*χ*^2^ = 25.6	<0.001
‌White matter hyperintensity (Fazekas grade 1: cap or pencil-thin lining)‌	2,264 (53.23%)	67 (36.02%)		
White matter hyperintensity (Fazekas grade 2: smooth halo or punctate confluence)‌	1,332 (31.32%)	72 (38.71%)		
White matter hyperintensity (Fazekas grade 3: irregular extension into deep white matter or large area confluence)‌	636 (14.95%)	47 (25.27%)		
‌Cerebral atrophy (defined as global cortical atrophy [GCA] score ≥2)‌	1,524 (35.83%)	46 (24.73%)	*χ*^2^ = 9.62	0.0019
Lacunar status (lacune)	1980 (46.56%)	120 (64.52%)	*χ*^2^ = 8.85	0.0030
Presence of cerebral microbleeds (CMB)	214 (5.03%)	12 (6.45%)	*χ*^2^ = 0.54	0.4620

**Figure 1:**
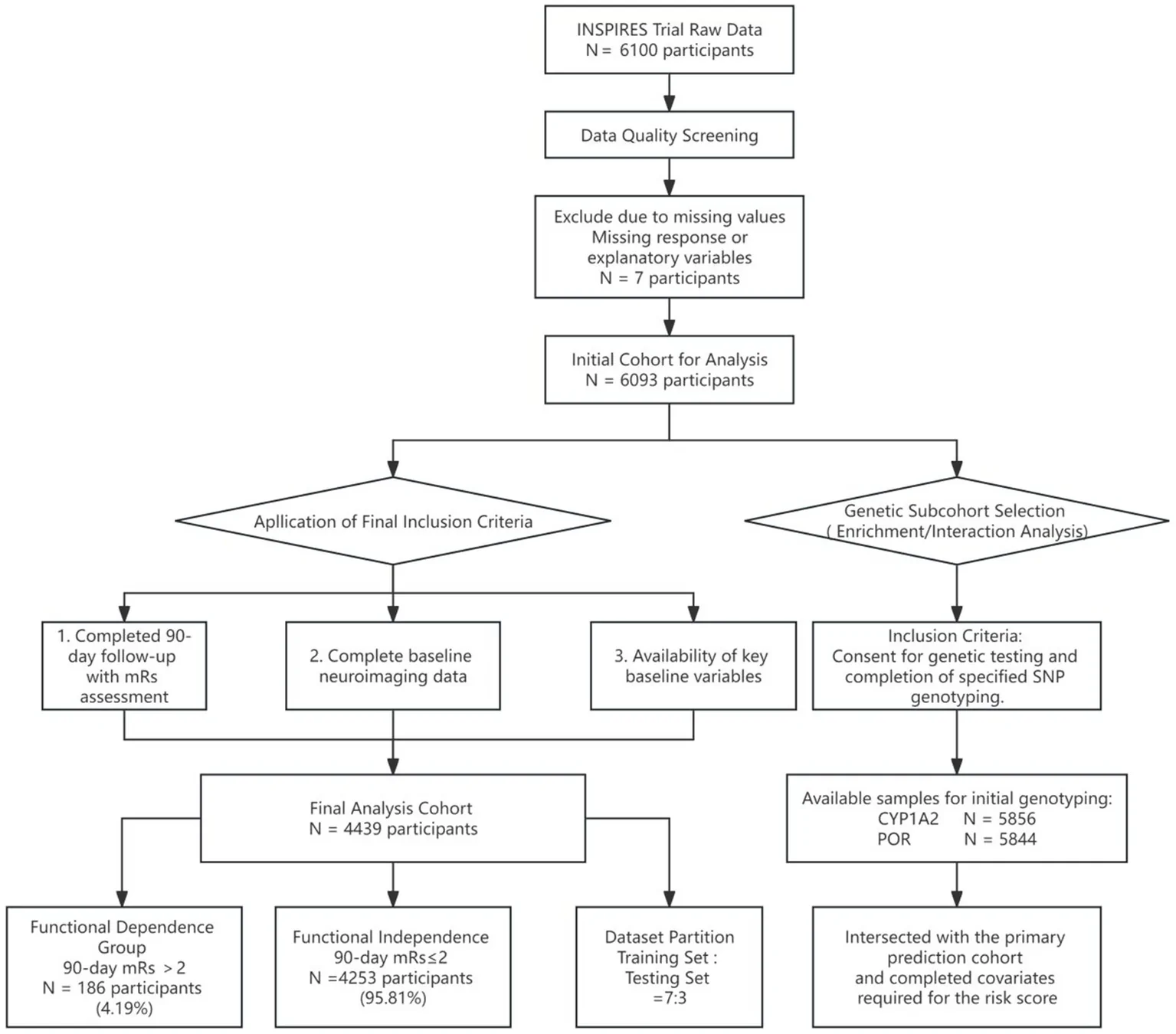
Patient screening flowchart.

### 3.2 Predictors selected by LASSO and final logistic regression model

Using LASSO logistic regression in the training set, a subset of baseline predictors with non-zero coefficients was selected. These predictors were then entered into an interpretable multivariable logistic regression model to estimate effect sizes. In the final model, older age, hypertension, diabetes, lower education level (Level 3 vs. Level 5 as the reference), and higher admission NIHSS score were independently associated with 90-day functional dependence (Table 2). Regarding education level, only the contrast between junior high school (Level 3) and university or above (Level 5) reached nominal statistical significance (OR = 0.637, *p* = 0.0484); no other education level contrasts (Level 1, 2, or 4 vs. Level 5) showed a significant association. Given the marginal nature of this finding and the possibility of residual confounding by age, this result should be interpreted with caution.

**Table 2:** A study on predicting functional dependence in 90-day period for patients with ischemic stroke: univariate and multivariate logistic regression analysis for 90-day functional dependence.

Predictor	Multivariate analysis (after LASSO selection)	*p*-value
	OR (95% CI)	
Age (per 1-year increase)	1.026 (1.005–1.047)	0.0112
Hypertension (H_HYPT01)	1.514 (1.038–2.207)	0.0302
Diabetes (H_DIAB01)	1.608 (1.174–2.204)	0.0028
Education level (B_EDUCATION)		
Level 1 vs. Level 5	0.411 (0.123–1.371)	0.1481
Level 2 vs. Level 5	0.716 (0.396–1.294)	0.2681
Level 3 vs. Level 5	0.637 (0.407–0.997)	0.0484
Level 4 vs. Level 5	0.793 (0.529–1.189)	0.2620
White matter hyperintensity (WMH)	1.165 (0.936–1.450)	0.1706
Cerebral atrophy (atrophy)	1.154 (0.787–1.693)	0.4631
Lacunar status (lacune)	1.113 (0.792–1.563)	0.5380
Admission NIHSS Score (A_NIHSS)	1.569 (1.432–1.718)	<0.0001
Pre-stroke mRS Score (A_MRS)	1.071 (0.831–1.382)	0.5962

*p* < 0.05 indicates a statistically significant difference.

### 3.3 Model performance, calibration, clinical utility, and interpretability

We compared the performance of four prediction algorithms. Logistic regression achieved the highest AUC point estimate (AUC = 0.727; 95% CI: 0.656–0.798) in the held-out test set, compared with XGBoost (AUC = 0.698; 95% CI: 0.622–0.774), random forest (AUC = 0.599; 95% CI: 0.527–0.671), and support vector machine (AUC = 0.495; 95% CI: 0.403–0.587) (Table 3; Figure 2A). The 95% confidence intervals overlapped across models, indicating that the observed AUC ranking lacked formal statistical support. Logistic regression was selected as the final model based on its interpretability and favorable calibration properties, rather than for statistical superiority. To provide a more objective evaluation under extreme class imbalance, we supplemented the traditional AUC with the area under the precision-recall curve (PR-AUC), the F1 score, and performance metrics at the Youden index-maximized threshold (Tables 3, 4). In the test set, logistic regression also demonstrated the best precision-recall trade-off for the minority class, achieving a PR-AUC of 0.412 (95% CI: 0.351–0.473), which surpassed XGBoost (0.386), random forest (0.312), and support vector machine (0.251). At the optimal probability threshold of approximately 0.067 (classifying patients as high-risk when predicted probability >6.7%), the logistic regression model achieved a sensitivity of 62.3%, specificity of 73.5%, positive predictive value of 9.2%, and negative predictive value of 97.8%. Furthermore, calibration assessment demonstrated good agreement between predicted and observed risks, with a calibration slope of 0.94 (95% CI: 0.81–1.07) and calibration-in-the-large of −0.04 (95% CI: −0.21 to 0.13) (Figure 2B). Decision curve analysis further confirmed that using this model for risk-based decision-making yields a net clinical benefit across clinically relevant threshold probabilities compared to “treat all” and “treat none” strategies (Figure 2C). Finally, SHAP-based interpretability analysis identified admission NIHSS, age > 65 years, and diabetes as the most influential features contributing to the predicted risk of functional dependence (Figure 2D).

**Table 3:** A study on predicting functional dependence in 90-day period for patients with ischemic stroke: final predictor variable of four prediction models on the test set.

Performance metric	Logistic regression	Random forest	Support vector machine	XGBoost
Feature importance (top 3)	A_NIHSS > age65 > H_DIAB01	A_NIHSS > age65 > H_DIAB01	A_NIHSS > H_DIAB01 > B_EDUCATION	A_NIHSS > H_HYPT01 > B_EDUCATION

**Figure 2:**
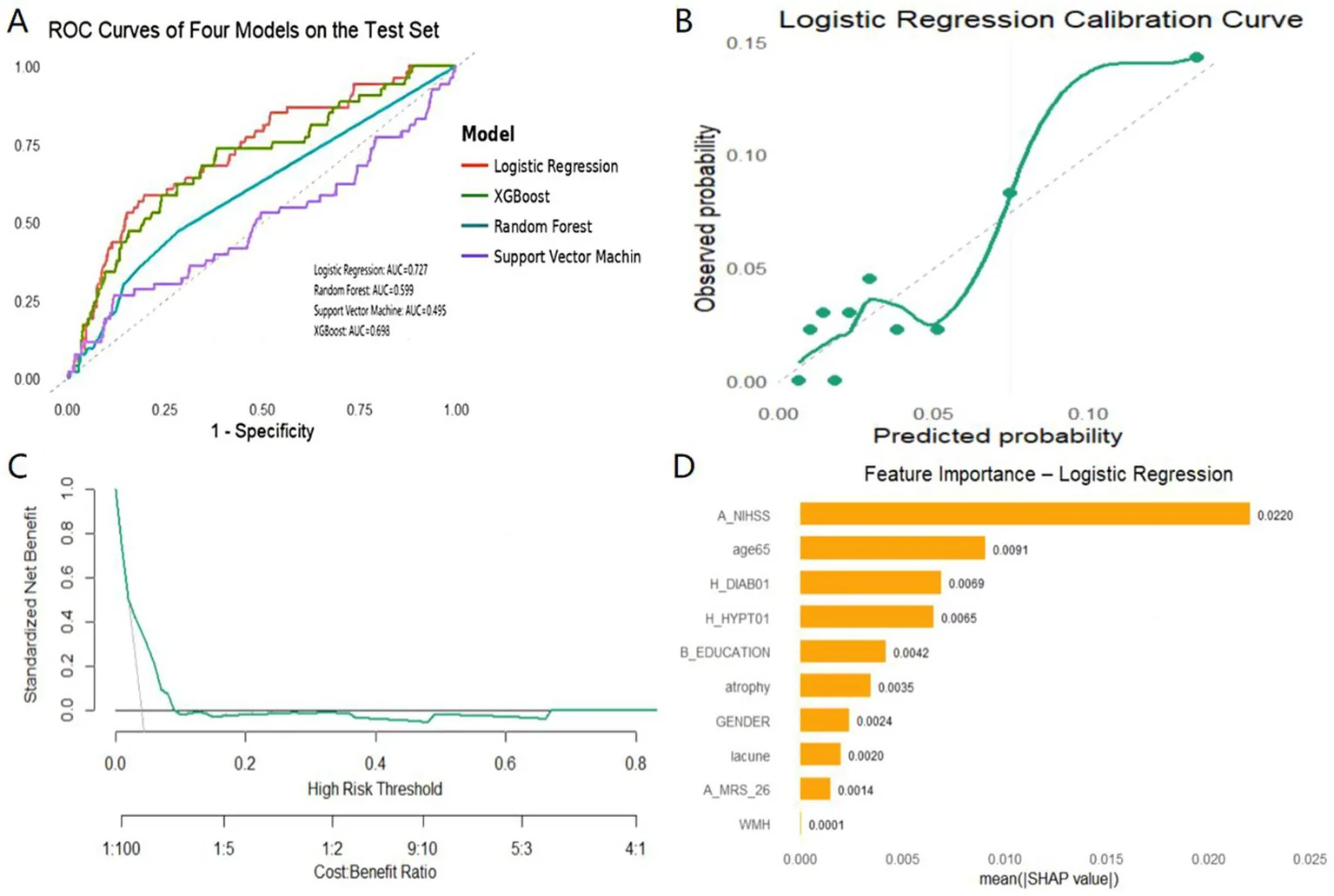
Comprehensive performance evaluation, clinical utility, and interpretability analysis of prediction models. **(A)** Comparison of Receiver Operating Characteristic (ROC) curves for four machine learning models on the test set; **(B)** Calibration curve of the logistic regression model; **(C)** Decision curve analysis based on the logistic regression model; **(D)** SHAP summary plot showing feature importance for predicting 90-day functional dependence with the logistic regression model.

**Table 4:** A study on predicting functional dependence in 90-day period for patients with ischemic stroke: performance comparison of four prediction models on the test set.

Model	AUC (95% CI)	Brier score	PR-AUC	*F*1-score	Optimal threshold	Performance at optimal threshold			
						Sensitivity (%)	Specificity (%)	PPV (%)	NPV (%)
Logistic regression	0.727 (0.656–0.798)	0.037	0.412	0.159	0.067	62.3	73.5	9.2	97.8
XGBoost	0.698 (0.622–0.774)	0.038	0.386	0.143	0.071	58.7	70.2	7.8	97.2
Random forest	0.599 (0.527–0.671)	0.040	0.312	0.105	0.055	51.4	65.8	6.1	96.5
Support vector machine	0.495 (0.403–0.587)	0.038	0.251	0.082	0.049	44.2	59.3	4.5	95.8

AUC, area under the receiver operating characteristic curve; PR-AUC, area under the precision-recall curve; PPV, positive predictive value; NPV, negative predictive value. The optimal threshold was determined by maximizing the Youden index (Youden index = sensitivity + specificity − 1). Due to the rarity of the outcome event (4.19%), none of the models could identify positive cases (sensitivity = 0) at the default threshold (0.5); therefore, performance is reported only at the optimal threshold. Logistic regression demonstrated superior sensitivity and specificity at the optimal threshold (predicted probability >6.7% classified as high risk) compared to other models.

### 3.4 Baseline risk stratification and association with antiplatelet strategy

Model-derived baseline risk stratification (prob_cat4) was strongly associated with 90-day functional dependence. After adjustment for age, hypertension, diabetes, and baseline NIHSS score, each increasing level of baseline risk category was associated with a higher odds of functional dependence (OR = 5.16; 95% CI, 3.81–6.99; *p* < 0.0001) (Table 5). In contrast, antiplatelet strategy (DAPT vs. single antiplatelet therapy) was not associated with reduced odds of functional dependence (OR = 1.00; 95% CI, 0.63–1.60; *p* = 1.000). No evidence of effect modification by baseline risk strata was observed (risk stratification × treatment interaction OR = 0.92; 95% CI, 0.50–1.69; *p* = 0.793).

**Table 5:** Comprehensive table of treatment heterogeneity analysis, core genotype distribution, and statistical tests: treatment-risk interaction analysis (*n* = 4,439).

Variable type	Specific variable	*β* coefficient (estimate)	Standard error	Wald *χ*^2^ value	*p*-value	OR (95% confidence interval)
Intercept	Intercept	−3.8110	0.1685	511.53	<0.0001	
Baseline variable	Baseline risk stratification (prob_cat4)	1.6409	0.1548	112.3201	<0.0001	5.16 (3.81~6.99)
Treatment variable	Dual antiplatelet therapy (dualAP)	5.03 × 10^−14^	0.2383	0.00	1.000	1.00 (0.63~1.60)
Interaction term	dualAP × prob_cat4	−0.0812	0.3097	0.07	0.793	0.92 (0.50~1.69)

prob_cat4 is the baseline risk stratification variable (risk level from low to high); dualAP is the treatment assignment variable (1 = received DAPT, 0 = received single antiplatelet therapy); the interaction term is used to test “the effect of baseline risk stratification on DAPT efficacy.” Statistical significance: *p* < 0.05 indicates a statistically significant difference; variables marked <0.0001 indicate strong significant associations. Data source: All values are from the multivariate logistic regression model output of the INSPIRES trial database.

### 3.5 Pharmacogenetic associations with functional outcome

A systematic candidate gene screening was performed on the full genotyping cohort (*n* ≈ 5,856), comprising all INSPIRES patients with available genotype data for the targeted loci. The screening panel included 107 SNPs across pathways related to clopidogrel metabolism, drug transport, and coagulation. Among all tested loci, CYP1A2 rs762551 and POR rs1057868 yielded the lowest *p*-values and were therefore selected for focused reporting. Complete results for all 107 SNPs are provided in Supplementary Table S5.

In the full genetic screening cohort (n ≈ 5,856, with 241 functional dependence events [4.11%]), we evaluated two prespecified candidate loci (CYP1A2 rs762551 and POR rs1057868) for associations with 90-day functional dependence and genotype enrichment patterns (Table 6; Figure 3). Baseline characteristics of genotyped versus non-genotyped patients were generally comparable (Supplementary Table S2), although the genetic subcohort was non-random and selection bias cannot be fully excluded. For CYP1A2 rs762551, the CC genotype was more frequent in the functional dependence group than in the functional independence group (23.65% vs. 16.28%), and genotype distribution differed between outcome groups (Pearson *χ*^2^ *p* = 0.0031; likelihood ratio *χ*^2^ *p* = 0.0042), while the trend test did not reach statistical significance (*p* = 0.0821). For POR rs1057868, the TC genotype was enriched in the functional dependence group (54.58% vs. 46.22%); associations were significant across *χ*^2^ tests (Pearson *χ*^2^ *p* = 0.0105; likelihood ratio *χ*^2^ *p* = 0.0091) with a significant Mantel–Haenszel trend (*p* = 0.0032), suggesting a possible allele-dosage pattern. Genotype enrichment plots further illustrated overrepresentation of CYP1A2 CC and POR TC genotypes among patients with poor functional outcomes (Figure 3).

**Table 6:** Comprehensive table of treatment heterogeneity analysis, core genotype distribution, and statistical tests: genotype distribution and statistical tests for CYP1A2 (rs762551) and POR (rs1057868).

Gene	Group	Functional independence group (mRS ≤ 2)	Functional dependence group (mRS > 2)	Total	Statistic (value)	*p*-value
CYP1A2 (rs762551)	AA genotype	2013 (35.85%)	90 (37.34%)	2,103		
	AC genotype	2,688 (47.87%)	94 (39.00%)	2,782		
	CC genotype	914 (16.28%)	57 (23.65%)	971		
	Total	5,615 (100.00%)	241 (100.00%)	5,856		
	Chi-square test	Pearson *χ*^2^			11.5458	0.0031
	Likelihood ratio Chi-square	Likelihood ratio *χ*^2^			10.9298	0.0042
	Mantel–Haenszel Chi-square	MH *χ*^2^			3.0223	0.0821
POR (rs1057868)	C genotype	2,219 (39.60%)	72 (30.00%)	2,291		
	TC genotype	2,590 (46.22%)	131 (54.58%)	2,721		
	T genotype	795 (14.19%)	37 (15.42%)	832		
	Total	5,604 (100.00%)	240 (100.00%)	5,844		
	Chi-square test	Pearson *χ*^2^			9.1112	0.0105
	Likelihood ratio Chi-square	Likelihood ratio *χ*^2^			9.3902	0.0091
	Mantel–Haenszel Chi-square	MH *χ*^2^			8.7123	0.0032

The CYP1A2 rs762551 CC genotype and the POR rs1057868 TC genotype were significantly enriched in the functional dependence group. The significance threshold after Bonferroni correction was set at *p* < 0.025, with both the Pearson *χ*^2^ *p*-value for CYP1A2 (*p* = 0.0031) and POR (*p* = 0.0105) falling below this threshold. Intra-group percentages were calculated as: (genotype count/total count of the corresponding outcome group) × 100%. The slight discrepancy in the total sample size between CYP1A2 and POR was due to varying rates of missing genotype data at each locus.

**Figure 3:**
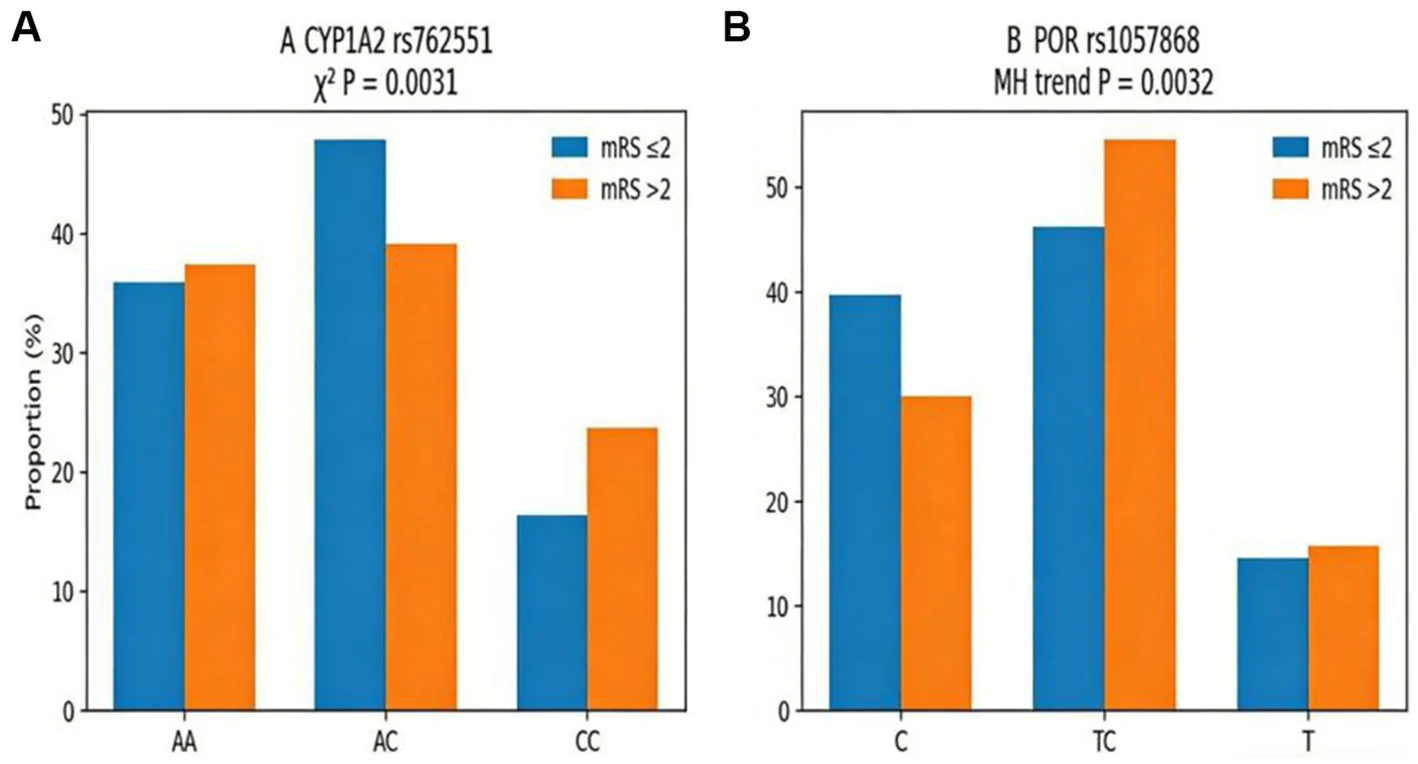
Enrichment of CYP1A2 and POR genotypes across different 90-day functional outcomes. **(A)** Distribution of CYP1A2 rs762551 genotypes between functional outcome groups. (**B)** Distribution of POR rs1057868 genotypes between functional outcome groups.

Hardy–Weinberg equilibrium testing in the functional independence group showed no deviation for either locus (CYP1A2 rs762551: *p* = 0.14; POR rs1057868: *p* = 0.22), confirming genotype quality control. Allele frequencies were consistent with East Asian reference populations from gnomAD (see Table 7).

**Table 7:** Comprehensive table of treatment heterogeneity analysis, core genotype distribution, and statistical tests: multivariable logistic regression analysis of genetic variants and 90-day functional dependence (adjusted for clinical covariates).

Gene	Genotype	Adjusted OR	95% CI	*p*-value
CYP1A2 rs762551	CC vs. AA + AC	1.72	1.18–2.51	0.004
POR rs1057868	TC vs. CC + TT	1.58	1.12–2.23	0.009

Covariates adjusted for included age, baseline NIHSS score, and history of diabetes. OR, odds ratio; CI, confidence interval. Bonferroni correction was applied for multiple comparisons, with a significance threshold set at *p* < 0.025.

In multivariable logistic regression adjusting for age, baseline NIHSS score, and diabetes, the association remained significant for both loci: CYP1A2 rs762551 CC genotype (adjusted OR = 1.72; 95% CI: 1.18–2.51; *p* = 0.004) and POR rs1057868 TC genotype (adjusted OR = 1.58; 95% CI: 1.12–2.23; *p* = 0.009), confirming that the observed genetic associations were not merely confounded by major clinical predictors (Supplementary Table S4).

## 4 Discussion

In secondary prevention after high-risk transient ischemic attack (TIA) and minor ischemic stroke, short-term dual antiplatelet therapy (DAPT) with aspirin plus clopidogrel is guideline-recommended and widely used in the acute phase (Karalija et al., 2022) (Lun et al., 2022; Chen et al., 2023; Khan et al., 2024). Large randomized trials have demonstrated reductions in recurrent ischemic events at the population level, supporting its role in contemporary guidelines (Gao et al., 2023; Elias et al., 2022). However, a clinically meaningful proportion of patients still experience unfavorable functional recovery at 90 days despite contemporary standard care (Lee Y. et al., 2024). In the INSPIRES trial, 186 patients (4.19%) remained functionally dependent at 90 days despite receiving standard DAPT. This outcome variability suggests that a “one-size-fits-all” approach based on population-average benefit may overlook baseline clinical and biological factors that shape prognosis and residual functional risk (Chalos et al., 2023). The core aim was not to re-evaluate the overall efficacy of DAPT, but to identify and quantify a clinically meaningful subgroup at high residual risk of 90-day functional dependence using baseline multimodal information. This approach moves the field from descriptive risk factors toward quantitative, individualized risk prediction and stratification based on patient-specific profiles.

This study adopted a two-stage analytic strategy: the first stage involved developing a clinical prediction model using multimodal baseline features to identify a high-risk subgroup for functional dependence; the second stage involved exploring, in an independent genotyped subcohort, whether specific pharmacogenetic variants are enriched among patients with unfavorable outcomes. Thus, genetic variants were not intended to be incorporated as predictors in the model; rather, they served as exploratory tools to understand the biology underlying the high-risk phenotype. This distinction is now reflected in the revised title and framing of the study.

Previous prognostic research has often examined single dimensions, such as neurological deficit severity (NIHSS) (Park et al., 2020; Landi et al., 2024; Georgakis et al., 2023; Lee et al., 2025) or isolated imaging markers (e.g., white matter hyperintensity burden or infarct volume) (Saber and Saver, 2020; Liu et al., 2024; Derraz et al., 2022). In contrast, our framework integrates multimodal baseline clinical and neuroimaging information into a single individualized prediction model. Using LASSO-based feature selection followed by multivariable logistic regression, we identified older age, hypertension, diabetes, and higher admission NIHSS score as independent predictors of 90-day functional dependence. Together, these factors delineate a clinically intuitive vulnerability profile reflecting reduced reserve (older age and potentially concomitant structural brain changes), higher systemic vascular risk burden (hypertension and diabetes), and greater acute injury severity (NIHSS). In such patients, the capacity to withstand ischemic injury and achieve functional recovery may be limited. Thus, beyond reiterating known predictors, the model quantifies their combined contribution at the individual level, supporting a shift from single-marker thinking toward an individualized risk configuration.

When comparing multiple algorithms, we observed that despite training and tuning random forest and XGBoost models with log-loss as the optimization objective, a parsimonious logistic regression model achieved the highest AUC point estimate in the held-out test set (Tang et al., 2025). However, the 95% confidence intervals for AUC overlapped substantially across models (logistic regression: 0.656–0.798; XGBoost: 0.622–0.774; random forest: 0.527–0.671; SVM: 0.403–0.587), indicating that the observed ranking lacked formal statistical support. Random forest (AUC = 0.599) and support vector machine (AUC = 0.495) performed significantly worse than logistic regression, a finding worth discussing. We attribute this difference primarily to extreme class imbalance (event rate of 4.19%), under which tree-based and kernel-based algorithms tend to shift their decision boundaries toward the majority class, even when class weights are applied. This observation aligns with prior literature, indicating that in clinical prediction scenarios involving rare events, logistic regression typically provides more stable probability estimates due to its linear additive structure and regularization. No formal pairwise statistical comparisons were performed between models, as the primary goal was not to identify a single “best” algorithm but to select a parsimonious, interpretable model for clinical application. Logistic regression, with its transparent structure and straightforward calibration, was selected as the final model for its interpretability and clinical utility, rather than for statistical superiority. This underscores a key translational principle: models intended for practice must be interpretable, trustworthy, and workflow-compatible, not merely technically sophisticated. A well-calibrated “white-box” model may have greater real-world impact than an opaque “black-box” model with marginally higher performance. For model interpretability, we used odds ratios and 95% confidence intervals as the primary tool, as logistic regression coefficients directly quantify predictor contributions in clinically meaningful units. SHAP visualizations, provided in Supplementary material, serve only as an auxiliary illustration of individual-level variation and do not replace the standard regression-based interpretation.

A key contribution of this study is the integration of prognostic prediction with evaluation of potential effect modification within a unified framework. Whereas many analyses emphasize average treatment effects (Beaulieu-Jones et al., 2023), we examined whether the association between antiplatelet strategy and functional dependence varied across model-derived baseline risk strata by testing an interaction term (treatment × risk stratification). We observed a strong gradient association between risk stratification and outcome (OR = 5.16 per increasing risk category), while the association between antiplatelet strategy and functional dependence did not differ across risk strata. These findings provide no evidence that baseline risk stratification modifies the association between antiplatelet strategy and 90-day functional dependence. Within this cohort, we did not observe differential associations of antiplatelet strategy across baseline risk levels for the functional dependence endpoint. This has implications for how prediction models may be used in clinical decision-making. When an analysis does not demonstrate effect modification for a given endpoint, the value of a prediction model may lie less in selecting among antiplatelet regimens and more in identifying patients at high residual risk who warrant intensified overall management (Andrés-Rebollo et al., 2025). Taken together, these results suggest that baseline vulnerability and injury severity are major determinants of functional prognosis in this dataset. Accordingly, individualized baseline risk prediction could help prioritize differentiated holistic management strategies and follow-up intensity (Shortreed et al., 2023). For patients identified as high risk, the stratification result itself has clinical alert value and may support earlier allocation of multidisciplinary resources, even if antiplatelet strategy differences are not apparent for this functional endpoint. A practical next question is which comprehensive interventions, beyond antiplatelet therapy, may improve functional recovery in these high-risk patients. Potential approaches include earlier rehabilitation planning, tighter comorbidity management, and prioritization for clinical studies evaluating neuroprotection, recovery-enhancing strategies, or other mechanisms (Zheng et al., 2026). For low-risk patients, risk stratification may help avoid unnecessary escalation of interventions and their associated adverse effects, including bleeding risk. Overall, risk-stratified allocation of preventive and rehabilitative resources may improve efficiency and align clinical intensity with patient need.

Notably, the multivariable logistic regression identified that patients with junior high school education (Level 3) had a lower adjusted risk of 90-day functional dependence compared to patients with university education or above (Level 5). However, this association was observed only for this single contrast, with no other education levels showing significant differences. After re-analyzing the underlying cohort data, we found this association to be largely driven by the strong correlation between age and education level within the functional dependence subgroup: among patients who ended up with 90-day functional dependence, those with university or above education had a substantially younger median age (58 years) compared to those with junior high school education (71 years). Given that older age is a well-established dominant risk factor for unfavorable functional recovery after stroke, this distribution likely leads to residual confounding even after conventional adjustment for age in the multivariable model. Therefore, we interpret this finding as an exploratory observation rather than a robust independent prognostic factor. Further dedicated investigations in larger, demographically balanced cohorts are warranted to systematically disentangle the complex interaction between age, socioeconomic status, educational attainment, and long-term functional prognosis after minor stroke/TIA.

We explored whether prespecified pharmacogenetic loci are associated with poor functional outcomes and enrichment among high-risk patients defined by the clinical prediction model. In a systematic screening of 107 candidate SNPs, CYP1A2 rs762551 and POR rs1057868 yielded the lowest *p*-values and were therefore selected for focused reporting (complete results for all 107 SNPs are provided in Supplementary Table S5). The model identified a highest-risk subgroup (Q4) with substantially elevated residual risk of functional dependence. In the genetic subcohort, CYP1A2 rs762551 CC and POR rs1057868 TC genotypes were enriched among patients with poor outcomes, supporting biologically plausible, hypothesis-generating links between metabolic and neuroregulatory pathways and functional recovery (Wang et al., 2025). In the genetic subcohort, CYP1A2 rs762551 CC and POR rs1057868 TC genotypes were enriched among patients with poor outcomes, supporting biologically plausible, hypothesis-generating links between metabolic and neuroregulatory pathways and functional recovery (Hingorani et al., 2023; van der Sangen et al., 2021). Mechanistically, rs762551 has been linked to variability in CYP1A2 inducibility; it is plausible that such variability could influence pathways relevant to clopidogrel bioactivation and clearance, although this study cannot establish causal pharmacokinetic mechanisms. (Capodanno and Angiolillo, 2023; Alkattan and Alsalameen, 2021). CYP1A2 and POR may indirectly modulate post-insult functional recovery through their regulatory effects on the metabolism of endogenous substrates such as the arachidonic acid signaling cascade, oxidative stress pathways, or molecular programs governing neural repair. Notably, the association between CYP1A2 rs762551 and functional outcome demonstrated some degree of model dependency: under the dominant model (CC vs. AA + AC), the adjusted OR was 1.72 (*p* = 0.008), whereas under the additive model (per additional C allele), the adjusted *p*-value (0.058) did not reach the Bonferroni-corrected significance threshold (0.0125). This pattern suggests that the effect of CYP1A2 rs762551 may be primarily driven by increased risk in CC homozygote carriers, rather than a linear allele-dosage effect. POR variants may influence post-stroke recovery through the encoded cytochrome P450 oxidoreductase, which serves as the essential electron donor to CYP enzymes, thereby affecting drug metabolism and endogenous signaling pathways; these pathways may also intersect with platelet-related mechanisms. POR variants may influence post-stroke recovery through the encoded cytochrome P450 oxidoreductase, which serves as the essential electron donor to CYP enzymes, thereby affecting drug metabolism and endogenous signaling pathways; these pathways may also intersect with platelet-related mechanisms (Yang et al., 2025). Given the observed associations and biological plausibility, these loci warrant further investigation in studies with pharmacokinetic/platelet function measurements and prospective validation.

Several limitations of this study should be acknowledged. First, the genetic subcohort was not randomly selected from the trial population, and although baseline characteristics were broadly comparable (Supplementary Table S2), selection bias cannot be fully excluded. This study involved two distinct genetic datasets: a full screening cohort (*n* ≈ 5,856) for the systematic SNP screening, and a randomly selected validation subcohort (*n* = 2,348) for baseline characteristic comparisons. Second, we did not genotype CYP2C19 major loss-of-function variants, which are known to influence clopidogrel metabolism; our focus on CYP1A2 and POR reflects the strongest signals emerging from a broader panel of candidate genes, and these findings should be interpreted as exploratory and hypothesis-generating rather than confirmatory. Third, the genetic analyses were conducted as a secondary investigation separate from the prediction model; we did not re-integrate genetic variants into the prediction pipeline, as this would conflate the distinct objectives of clinical risk stratification and biological mechanism exploration. Fourth, our prediction model was developed using complete-case analysis. While missing data proportions were low (Supplementary Table S1), we cannot entirely rule out selection bias if missingness was not completely at random. However, sensitivity analyses using multiple imputation yielded results consistent with the main findings (age: adjusted OR = 1.025, *p* = 0.015; NIHSS: adjusted OR = 1.55, *p* < 0.001; diabetes: adjusted OR = 1.58, *p* = 0.004), mitigating this concern.

Notably, CYP2C19 genotyping was not performed in the present cohort, and the functional role of the investigated CYP1A2 variant has not been experimentally validated in dedicated mechanistic studies. Accordingly, all findings reported here should be exclusively interpreted for hypothesis-generating purposes, and no direct clinical guidance for antiplatelet agent selection or dosage adjustment should be derived from these results.

These results should be interpreted as hypothesis-generating and do not support genotype-based treatment changes in routine practice. Instead, they motivate future prospective studies to evaluate whether genotype-informed strategies (e.g., alternative P2Y12 inhibitors) or adjunctive recovery-focused interventions improve outcomes in high-risk patients (Capodanno and Angiolillo, 2023; Pereira et al., 2024). In summary, our framework enables quantifiable individualized risk stratification for functional dependence and provides pharmacogenetic signals associated with poor outcomes from the perspectives of metabolism- and neuroregulation-related biology. This work supports a pragmatic shift from population-average risk assessment toward data-driven, interpretable risk stratification, while highlighting candidate biological features that merit prospective validation.

## 5 Conclusion

In this secondary analysis of the INSPIRES trial, an interpretable clinical prediction model integrating multimodal baseline clinical and neuroimaging features identified a high-risk subgroup comprising 25% of patients that accounted for a disproportionate burden of 90-day functional dependence after minor stroke/TIA. Logistic regression was selected as the final model based on its interpretability and calibration properties, rather than for statistical superiority over other machine learning approaches. Key independent predictors included age >65 years, admission NIHSS score, hypertension, and diabetes. In separate exploratory analyses of 107 candidate SNPs, CYP1A2 rs762551 and POR rs1057868 variants were associated with poor outcomes and enriched in this subgroup, generating pharmacogenetic hypotheses for future investigation. Although antiplatelet strategy did not modify risk across strata, baseline risk stratification strongly predicted functional prognosis. These findings support individualized risk assessment to prioritize high-risk patients for intensified multidisciplinary management and future trials of tailored secondary prevention.

## Data Availability

The original contributions presented in the study are included in the article/Supplementary material, further inquiries can be directed to the corresponding author.
