# Supplementary material for: A clinical prediction model for 90-day functional dependence after minor stroke/TIA with exploratory pharmacogenetic associations: a post-hoc analysis of the INSPIRES trial
Source: Front Aging Neurosci. 2026 Oct 14;18:1922100. doi: 10.3389/fnagi.2026.1922100 (PMC13713692; doi:10.3389/fnagi.2026.1922100)
Supplement: Supplementary file 1 [file Data_Sheet_1.docx]

**Supplementary Table S1. Missing Data for Baseline Variables**

| **Variable** | **Total Cases** | **Missing Cases** | **Missing Proportion (%)** |
| --- | --- | --- | --- |
| Age | 4439 | 0 | 0.0 |
| Sex | 4439 | 0 | 0.0 |
| History of Hypertension | 4439 | 0 | 0.0 |
| History of Diabetes | 4439 | 0 | 0.0 |
| Admission NIHSS Score | 4439 | 0 | 0.0 |
| Premorbid mRS Score | 4439 | 0 | 0.0 |
| Education Level | 4439 | 0 | 0.0 |
| Insurance Type | 4439 | 0 | 0.0 |
| WMH Grade | 4439 | 42 | 0.9 |
| Brain Atrophy | 4439 | 51 | 1.1 |
| Lacune Status | 4439 | 38 | 0.9 |
| Cerebral Microbleeding | 4439 | 186 | 4.2 |
| Note：Except for cerebral microbleeds, the proportion of missing data for all variables was less than 2%. The missing rate for cerebral microbleeds was slightly higher (4.2%), as some centers did not routinely perform SWI/GRE-T2* sequences. | | | |

**Supplementary Table S2. Comparison of Baseline Characteristics Between the Genotyped Subcohort and Non-genotyped Patients**

| **Characteristic** | **Genotyped Subcohort (n = 2,348)** | **Non-genotyped Patients (n = 2,091)** | **Test Statistic** | **P value** |
| --- | --- | --- | --- | --- |
| Age (years), mean ± SD | 66.1 ± 9.8 | 64.8 ± 10.2 | t = 1.55 | 0.12 |
| Age > 65 years, n (%) | 1,198 (51.0) | 1,032 (49.4) | χ² = 1.17 | 0.28 |
| Male, n (%) | 1,477 (62.9) | 1,343 (64.2) | χ² = 0.83 | 0.36 |
| Hypertension, n (%) | 1,596 (68.0) | 1,414 (67.6) | χ² = 0.06 | 0.80 |
| Diabetes, n (%) | 648 (27.6) | 562 (26.9) | χ² = 0.29 | 0.59 |
| Admission NIHSS score, median (IQR) | 2 (0–3) | 2 (0–3) | Z = 0.80 | 0.42 |
| Pre-morbid mRS score, median (IQR) | 0 (0–0) | 0 (0–0) | Z = 0.41 | 0.68 |
| Time from onset to randomization ≤ 24 hours, n (%) | 1,452 (61.8) | 1,286 (61.5) | χ² = 0.05 | 0.83 |
| 90-day functional dependence (mRS > 2), n (%) | 144 (6.1) | 104 (5.0) | χ² = 2.88 | 0.09 |
| Note：Comparisons between groups were performed using the two-sample t-test for normally distributed continuous variables, the Wilcoxon rank-sum test for non-normally distributed continuous variables, or the Pearson χ² test for categorical variables. t, Z, and χ² denote the test statistics for the t-test, Wilcoxon rank-sum test, and Pearson chi-square test, respectively. P < 0.05 was considered statistically significant. The baseline characteristics were generally comparable between the two groups (all P > 0.05), indicating a low risk of selection bias; however, the non-random nature of the genetic subcohort should be noted. | | | | |

**Supplementary Table S3. Confusion Matrices and Classification Performance of Each Model at the Optimal Threshold**

| **Model** | **Optimal Threshold** | **TP** | **FP** | **FN** | **TN** | **Sensitivity (%)** | **Specificity (%)** | **PPV (%)** | **NPV (%)** | **F1 Score** |
| --- | --- | --- | --- | --- | --- | --- | --- | --- | --- | --- |
| Logistic Regression | 0.067 | 46 | 350 | 28 | 975 | 62.3 | 73.5 | 9.2 | 97.8 | 0.159 |
| XGBoost | 0.071 | 43 | 395 | 31 | 930 | 58.7 | 70.2 | 7.8 | 97.2 | 0.143 |
| Random Forest | 0.055 | 38 | 453 | 36 | 872 | 51.4 | 65.8 | 6.1 | 96.5 | 0.105 |
| Support Vector Machine | 0.049 | 33 | 539 | 41 | 786 | 44.2 | 59.3 | 4.5 | 95.8 | 0.082 |
| Note：TP, true positive (actual dependence, predicted dependence); FP, false positive (actual non-dependence, predicted dependence); FN, false negative (actual dependence, predicted non-dependence); TN, true negative (actual non-dependence, predicted non-dependence). The total sample size of the test set was 1,399, including 74 cases of functional dependence (positive class), accounting for 5.29%. All metrics were calculated based on the optimal threshold that maximized the Youden index. PPV, positive predictive value; NPV, negative predictive value. Due to the extremely low event rate, the PPV was generally low across all models; however, the NPV exceeded 95% for all models, suggesting a high clinical value in ruling out low-risk patients. Logistic regression demonstrated superior sensitivity and F1 score at the optimal threshold compared to other models. | | | | | | | | | | |

**Supplementary Table S4. Multivariable Logistic Regression Analysis of Genetic Variants and 90-day Functional Dependence**

| **Gene/Locus** | **Genetic Model** | **Genotype Comparison** | **Adjusted OR** | **95% CI** | **P value** | **Adjusted P value aa** |
| --- | --- | --- | --- | --- | --- | --- |
| CYP1A2 rs762551 | Dominant | CC vs. AA+AC | 1.72 | 1.18 - 2.51 | 0.004 | 0.008 |
| CYP1A2 rs762551 | Additive | Per additional C allele | 1.31 | 1.03 - 1.67 | 0.029 | 0.058 |
| POR rs1057868 | Dominant | TC vs. CC+TT | 1.58 | 1.12 - 2.23 | 0.009 | 0.018 |
| POR rs1057868 | Additive | Per additional T allele | 1.28 | 1.01 - 1.62 | 0.041 | 0.082 |
| Note：Covariates included age, baseline NIHSS score, and history of diabetes. OR = odds ratio; CI = confidence interval. Adjusted P value aa were corrected for multiple comparisons using the Bonferroni method (four genetic model-locus combinations tested; correction factor = 4; significance threshold, P < 0.0125). CYP1A2 CC (dominant model) and POR TC (dominant model) remained significant after adjustment (adjusted P < 0.0125), indicating that these signals are not solely attributable to multiple testing. | | | | | | |

## Supplementary Table S5. Association of 107 Candidate SNPs with 90-Day Functional Dependence (Complete Screening Results)

|  | SNP | Gene | Events (mRS>2) n/N (%) | Non-events (mRS≤2) n/N (%) | χ² (df) | P-value | Bonferroni-adjusted P† |
| --- | --- | --- | --- | --- | --- | --- | --- |
| 1 | rs762551 | CYP1A2 | 57/241 (23.65) | 914/5,615 (16.28) | 11.55 (2) | 0.0031 | 0.332 |
| 2 | rs1057868 | POR | 131/240 (54.58) | 2,590/5,604 (46.22) | 9.11 (2) | 0.0105 | 1.000 |
| 3 | rs12248560 | CYP2C19 | 5/238 (2.10) | 113/5,586 (2.02) | 10.77 (2)‡ | 0.0046‡ | 0.492 |
| 4 | rs1080983 | CYP2D6 | 240/5,842 (4.11) | 5,602/5,842 (95.89) | 11.61 (3) | 0.0088 | 0.942 |
| 5 | rs3818822 | CHIA | 241/5,864 (4.11) | 5,623/5,864 (95.89) | 7.36 (2) | 0.0253 | 1.000 |
| 6 | rs2242480 | CYP3A4 | 241/5,857 (4.11) | 5,616/5,857 (95.89) | 6.35 (2) | 0.0418 | 1.000 |
| 7 | rs2107595 | HDAC9 | 241/5,860 (4.11) | 5,619/5,860 (95.89) | 5.93 (2) | 0.0517 | 1.000 |
| 8 | rs8192719 | CYP2B6 | 241/5,865 (4.11) | 5,624/5,865 (95.89) | 5.37 (2) | 0.0683 | 1.000 |
| 9 | rs1799722 | BDKRB2 | 236/5,805 (4.07) | 5,569/5,805 (95.93) | 5.45 (2) | 0.0656 | 1.000 |
| 10 | rs2302429 | POR | 240/5,852 (4.10) | 5,612/5,852 (95.90) | 5.77 (2) | 0.0559 | 1.000 |
| 11 | rs3745274 | CYP2B6 | 240/5,859 (4.10) | 5,619/5,859 (95.90) | 5.18 (2) | 0.0751 | 1.000 |
| 12 | rs1105783 | NCOR2 | 241/5,859 (4.11) | 5,618/5,859 (95.89) | 5.07 (2) | 0.0795 | 1.000 |
| 13 | rs4244285 | CYP2C19 | 239/5,832 (4.10) | 5,593/5,832 (95.90) | 4.93 (2) | 0.0852 | 1.000 |
| 14 | rs12579302 | ATP2B1 | 240/5,860 (4.10) | 5,620/5,860 (95.90) | 4.58 (2) | 0.1014 | 1.000 |
| 15 | rs1472122 | MED12L | 241/5,860 (4.11) | 5,619/5,860 (95.89) | 4.50 (2) | 0.1052 | 1.000 |
| 16 | rs6536024 | FGA | 239/5,847 (4.09) | 5,608/5,847 (95.91) | 4.25 (2) | 0.1195 | 1.000 |
| 17 | rs2316757 | RPRML | 240/5,860 (4.10) | 5,620/5,860 (95.90) | 4.08 (2) | 0.1298 | 1.000 |
| 18 | rs28365085 | CYP3A5 | 240/5,862 (4.09) | 5,622/5,862 (95.91) | 4.12 (2)‡ | 0.1274‡ | 1.000 |
| 19 | rs12445022 | ZCCHC14 | 241/5,864 (4.11) | 5,623/5,864 (95.89) | 4.02 (2) | 0.1339 | 1.000 |
| 20 | rs833509 | DYM | 241/5,862 (4.11) | 5,621/5,862 (95.89) | 3.59 (2) | 0.1659 | 1.000 |
| 21 | rs2455132 | PRDM16 | 241/5,862 (4.11) | 5,621/5,862 (95.89) | 3.63 (3) | 0.3042 | 1.000 |
| 22 | rs4693075 | COQ2 | 240/5,860 (4.10) | 5,620/5,860 (95.90) | 3.02 (3) | 0.3881 | 1.000 |
| 23 | rs2286823 | POR | 239/5,850 (4.09) | 5,611/5,850 (95.91) | 9.57 (5) | 0.0885 | 1.000 |
| 24 | rs4803419 | CYP2B6 | 241/5,861 (4.11) | 5,620/5,861 (95.89) | 3.28 (2) | 0.1943 | 1.000 |
| 25 | rs16998073 | FGF5 | 240/5,858 (4.10) | 5,618/5,858 (95.90) | 3.24 (2) | 0.1979 | 1.000 |
| 26 | rs42035 | CDK6 | 241/5,863 (4.11) | 5,622/5,863 (95.89) | 3.25 (2) | 0.1971 | 1.000 |
| 27 | rs7070678 | SVIL | 241/5,863 (4.11) | 5,622/5,863 (95.89) | 2.83 (2) | 0.2432 | 1.000 |
| 28 | rs7910521 | SVIL | 241/5,862 (4.11) | 5,621/5,862 (95.89) | 2.97 (2) | 0.2265 | 1.000 |
| 29 | rs7913801 | SVIL | 241/5,862 (4.11) | 5,621/5,862 (95.89) | 3.01 (2) | 0.2216 | 1.000 |
| 30 | rs1574430 | SLC22A7 | 241/5,853 (4.12) | 5,612/5,853 (95.88) | 2.98 (2) | 0.2259 | 1.000 |
| 31 | rs7859362 | CDKN2B-AS1 | 239/5,852 (4.08) | 5,613/5,852 (95.92) | 3.02 (2) | 0.2209 | 1.000 |
| 32 | rs56260937 | PEAR1 | 241/5,863 (4.11) | 5,622/5,863 (95.89) | 2.91 (2) | 0.2339 | 1.000 |
| 33 | rs2244613 | CES1 | 241/5,860 (4.11) | 5,619/5,860 (95.89) | 2.92 (2) | 0.2322 | 1.000 |
| 34 | rs4803418 | CYP2B6 | 240/5,861 (4.09) | 5,621/5,861 (95.91) | 3.07 (2) | 0.2158 | 1.000 |
| 35 | rs6722806 | THADA | 241/5,860 (4.11) | 5,619/5,860 (95.89) | 3.12 (2) | 0.2101 | 1.000 |
| 36 | rs13148045 | PCDH18 | 239/5,841 (4.09) | 5,602/5,841 (95.91) | 3.05 (2) | 0.2174 | 1.000 |
| 37 | rs11265613 | IL6R | 240/5,845 (4.11) | 5,605/5,845 (95.89) | 3.00 (2) | 0.2232 | 1.000 |
| 38 | rs1573644 | FURIN | 240/5,853 (4.10) | 5,613/5,853 (95.90) | 2.90 (2) | 0.2344 | 1.000 |
| 39 | rs4487481 | TRIM36 | 241/5,863 (4.11) | 5,622/5,863 (95.89) | 2.83 (2) | 0.2424 | 1.000 |
| 40 | rs11078685 | POLR2A | 241/5,860 (4.11) | 5,619/5,860 (95.89) | 2.64 (2) | 0.2672 | 1.000 |
| 41 | rs662799 | APOA5 | 241/5,860 (4.11) | 5,619/5,860 (95.89) | 2.66 (2) | 0.2639 | 1.000 |
| 42 | rs7687767 | NOA1 | 240/5,808 (4.13) | 5,568/5,808 (95.87) | 2.76 (2) | 0.2521 | 1.000 |
| 43 | rs10826650 | SVIL | 241/5,863 (4.11) | 5,622/5,863 (95.89) | 2.58 (2) | 0.2757 | 1.000 |
| 44 | rs1878406 | EDNRA | 241/5,863 (4.11) | 5,622/5,863 (95.89) | 2.58 (2) | 0.2756 | 1.000 |
| 45 | rs55983834 | SH3PXD2A | 241/5,865 (4.11) | 5,624/5,865 (95.89) | 2.55 (2) | 0.2792 | 1.000 |
| 46 | rs60401382 | HTRA1 | 240/5,861 (4.09) | 5,621/5,861 (95.91) | 2.54 (2) | 0.2815 | 1.000 |
| 47 | rs28360521 | CYP2D6 | 239/5,845 (4.09) | 5,606/5,845 (95.91) | 2.07 (2) | 0.3549 | 1.000 |
| 48 | rs2108622 | CYP4F2 | 240/5,861 (4.09) | 5,621/5,861 (95.91) | 2.38 (2) | 0.3046 | 1.000 |
| 49 | rs7304841 | PDE3A | 240/5,859 (4.10) | 5,619/5,859 (95.90) | 2.39 (2) | 0.3034 | 1.000 |
| 50 | rs11042273 | TMEM41B | 238/5,831 (4.08) | 5,593/5,831 (95.92) | 3.91 (2) | 0.1417 | 1.000 |
| 51 | rs2032582 | ABCB1 | 240/5,851 (4.10) | 5,611/5,851 (95.90) | 3.96 (5) | 0.5558 | 1.000 |
| 52 | rs1126643 | ITGA2 | 241/5,861 (4.11) | 5,620/5,861 (95.89) | 2.21 (2) | 0.3315 | 1.000 |
| 53 | rs4986893 | CYP2C19 | 241/5,863 (4.11) | 5,622/5,863 (95.89) | 2.22 (2) | 0.3291 | 1.000 |
| 54 | rs2487928 | JCAD | 241/5,862 (4.11) | 5,621/5,862 (95.89) | 2.27 (2) | 0.3218 | 1.000 |
| 55 | rs2663905 | MESDC1 | 241/5,861 (4.11) | 5,620/5,861 (95.89) | 1.95 (2) | 0.3767 | 1.000 |
| 56 | rs2231142 | ABCG2 | 241/5,865 (4.11) | 5,624/5,865 (95.89) | 1.98 (2) | 0.3709 | 1.000 |
| 57 | rs7973143 | ANKRD33 | 240/5,855 (4.10) | 5,615/5,855 (95.90) | 1.87 (2) | 0.3925 | 1.000 |
| 58 | rs9515201 | COL4A2 | 240/5,858 (4.10) | 5,618/5,858 (95.90) | 4.29 (2) | 0.1173 | 1.000 |
| 59 | rs28673728 | INPP5B | 240/5,861 (4.09) | 5,621/5,861 (95.91) | 1.84 (2) | 0.3992 | 1.000 |
| 60 | rs557675 | OVOL1 | 233/5,759 (4.05) | 5,526/5,759 (95.95) | 1.65 (2) | 0.4376 | 1.000 |
| 61 | rs8014986 | HHIPL1 | 241/5,865 (4.11) | 5,624/5,865 (95.89) | 1.47 (2) | 0.4803 | 1.000 |
| 62 | rs1487504 | BNC2 | 241/5,861 (4.11) | 5,620/5,861 (95.89) | 1.45 (2) | 0.4849 | 1.000 |
| 63 | rs1412444 | LIPA | 241/5,862 (4.11) | 5,621/5,862 (95.89) | 1.76 (2) | 0.4146 | 1.000 |
| 64 | rs2501966 | CENPQ | 241/5,862 (4.11) | 5,621/5,862 (95.89) | 1.76 (2) | 0.4141 | 1.000 |
| 65 | rs1045642 | ABCB1 | 240/5,842 (4.11) | 5,602/5,842 (95.89) | 1.69 (2) | 0.4296 | 1.000 |
| 66 | rs748431 | FGD5 | 240/5,858 (4.10) | 5,618/5,858 (95.90) | 1.70 (2) | 0.4280 | 1.000 |
| 67 | rs2359171 | ZFHX3 | 241/5,861 (4.11) | 5,620/5,861 (95.89) | 1.69 (2) | 0.4306 | 1.000 |
| 68 | rs776746 | CYP3A5 | 241/5,863 (4.11) | 5,622/5,863 (95.89) | 1.61 (2) | 0.4466 | 1.000 |
| 69 | rs680084 | PRRX1 | 240/5,845 (4.11) | 5,605/5,845 (95.89) | 1.51 (2) | 0.4703 | 1.000 |
| 70 | rs7800053 | EVX1 | 241/5,863 (4.11) | 5,622/5,863 (95.89) | 1.52 (2) | 0.4684 | 1.000 |
| 71 | rs2405068 | PTCH1 | 241/5,856 (4.12) | 5,615/5,856 (95.88) | 1.48 (2) | 0.4766 | 1.000 |
| 72 | rs429358 | APOE | 234/5,706 (4.10) | 5,472/5,706 (95.90) | 0.79 (2) | 0.6722 | 1.000 |
| 73 | rs7412 | APOE | 237/5,809 (4.08) | 5,572/5,809 (95.92) | 0.22 (2) | 0.8968 | 1.000 |
| 74 | rs662 | PON1 | 241/5,865 (4.11) | 5,624/5,865 (95.89) | 0.32 (2) | 0.8533 | 1.000 |
| 75 | rs854560 | PON1 | 241/5,863 (4.11) | 5,622/5,863 (95.89) | 0.59 (2) | 0.7455 | 1.000 |
| 76 | rs1128503 | ABCB1 | 232/5,664 (4.10) | 5,432/5,664 (95.90) | 1.39 (2) | 0.5000 | 1.000 |
| 77 | rs20455 | KIF6 | 241/5,860 (4.11) | 5,619/5,860 (95.89) | 0.98 (2) | 0.6117 | 1.000 |
| 78 | rs5918 | ITGB3 | 239/5,854 (4.08) | 5,615/5,854 (95.92) | 0.70 (2) | 0.7039 | 1.000 |
| 79 | rs6065 | ITGA2 | 241/5,861 (4.11) | 5,620/5,861 (95.89) | 0.75 (2) | 0.6885 | 1.000 |
| 80 | rs701265 | P2RY1 | 241/5,861 (4.11) | 5,620/5,861 (95.89) | 1.15 (2) | 0.5623 | 1.000 |
| 81 | rs649129 | ABO | 238/5,839 (4.08) | 5,601/5,839 (95.92) | 1.12 (2) | 0.5726 | 1.000 |
| 82 | rs854572 | PON1 | 241/5,859 (4.11) | 5,618/5,859 (95.89) | 1.07 (2) | 0.5851 | 1.000 |
| 83 | rs2306283 | SLCO1B1 | 241/5,861 (4.11) | 5,620/5,861 (95.89) | 1.08 (2) | 0.5837 | 1.000 |
| 84 | rs4148727 | — | 240/5,851 (4.10) | 5,611/5,851 (95.90) | 0.76 (2) | 0.6854 | 1.000 |
| 85 | rs10120688 | CDKN2B-AS1 | 241/5,864 (4.11) | 5,623/5,864 (95.89) | 0.27 (2) | 0.8750 | 1.000 |
| 86 | rs10111852 | PTK2 | 241/5,865 (4.11) | 5,624/5,865 (95.89) | 0.20 (2) | 0.9056 | 1.000 |
| 87 | rs12426667 | HOXC4 | 241/5,865 (4.11) | 5,624/5,865 (95.89) | 0.10 (2) | 0.9492 | 1.000 |
| 88 | rs4918758 | CYP2C9 | 241/5,865 (4.11) | 5,624/5,865 (95.89) | 0.05 (2) | 0.9777 | 1.000 |
| 89 | rs1549758 | NOS3 | 240/5,857 (4.10) | 5,617/5,857 (95.90) | 0.18 (2) | 0.9142 | 1.000 |
| 90 | rs4149056 | SLCO1B1 | 240/5,862 (4.09) | 5,622/5,862 (95.91) | 0.22 (2) | 0.8960 | 1.000 |
| 91 | rs2738158 | DEFB1 | 241/5,840 (4.13) | 5,599/5,840 (95.87) | 0.45 (2) | 0.7985 | 1.000 |
| 92 | rs880315 | CASZ1 | 241/5,862 (4.11) | 5,621/5,862 (95.89) | 0.84 (2) | 0.6564 | 1.000 |
| 93 | rs4298492 | EBF2 | 241/5,859 (4.11) | 5,618/5,859 (95.89) | 0.82 (2) | 0.6627 | 1.000 |
| 94 | rs2351524 | NBEAL1 | 241/5,863 (4.11) | 5,622/5,863 (95.89) | 0.90 (2) | 0.6389 | 1.000 |
| 95 | rs11691032 | FIGN | 237/5,834 (4.06) | 5,597/5,834 (95.94) | 0.48 (2) | 0.7849 | 1.000 |
| 96 | rs1799983 | NOS3 | 241/5,859 (4.11) | 5,618/5,859 (95.89) | 0.14 (2) | 0.9310 | 1.000 |
| 97 | rs730012 | LTC4S | 241/5,859 (4.11) | 5,618/5,859 (95.89) | 0.16 (3) | 0.9834 | 1.000 |
| 98 | rs2901029 | ATP1B1 | 240/5,861 (4.09) | 5,621/5,861 (95.91) | 0.67 (2) | 0.7163 | 1.000 |
| 99 | rs2397816 | LINC00924 | 240/5,861 (4.09) | 5,621/5,861 (95.91) | 0.39 (2) | 0.8237 | 1.000 |
| 100 | rs2768759 | PEAR1 | 240/5,861 (4.09) | 5,621/5,861 (95.91) | 0.76 (2) | 0.6849 | 1.000 |
| 101 | rs114798023 | THAP5 | 241/5,859 (4.11) | 5,618/5,859 (95.89) | 0.89 (2) | 0.6420 | 1.000 |
| 102 | rs17148926 | LOC100505841 | 240/5,853 (4.10) | 5,613/5,853 (95.90) | 0.41 (2) | 0.8167 | 1.000 |
| 103 | rs17517928 | FN1 | 241/5,865 (4.11) | 5,624/5,865 (95.89) | 0.15 (2) | 0.9276 | 1.000 |
| 104 | rs56337013 | CYP2C19 | 241/5,865 (4.11) | 5,624/5,865 (95.89) | 0.04 (1) | 0.8360 | 1.000 |
| 105 | rs41291556 | CYP2C19 | — | — | — | — | — |
| 106 | rs28371685 | CYP2C9 | — | — | — | — | — |
| 107 | rs2877984 | LAMC1 | — | — | — | — | — |

† Bonferroni correction for 107 tests: significance threshold α = 0.05/107 ≈ 0.00047. No SNP reached this threshold.

‡ For SNPs with expected cell counts <5 (marked with WARNING), the chi-square test may be invalid; Fisher's exact test results from the original SAS output should be interpreted instead.

Note onSNP Selection for Main Manuscript Reporting:

Among the 107 candidate SNPs systematically screened, rs762551 (CYP1A2) and rs1057868 (POR) were selected for focused reporting in the main manuscript based on the following criteria:

(1) Lowest P-values in the screening panel – These two loci yielded the smallest P-values among all tested SNPs (rs762551: P=0.0031; rs1057868: P=0.0105), representing the strongest association signals detected in this exploratory screening.

(2) Biological plausibility – CYP1A2 is a phase I metabolizing enzyme that contributes to clopidogrel oxidative metabolism, and POR encodes the essential electron donor for all cytochrome P450 enzymes, positioning both genes within biologically relevant pathways for antiplatelet drug response and post-stroke recovery.

(3) Consistency of genetic association patterns – Both loci demonstrated consistent genotype enrichment patterns (CYP1A2 CC and POR TC genotypes were overrepresented in the functional dependence group), with POR additionally showing a significant allele-dosage trend (P=0.0032).

(4) Exclusion of other SNPs – The remaining 105 SNPs were not selected for main-text reporting because: (a) none remained significant after Bonferroni correction for multiple testing (adjusted P > 0.47 for all); (b) several nominally significant SNPs (e.g., rs12248560, rs1080983) had expected cell counts <5, rendering chi-square test results unreliable; (c) many showed P-values >0.05 with no consistent genotype-enrichment patterns; and (d) several loci were monomorphic in this cohort and provided no informative genetic variation.

All findings from this screening are exploratory and hypothesis-generating in nature, requiring independent validation in future well-powered studies before any definitive conclusions can be drawn. The complete results of all 107 SNPs are provided here for full transparency to exclude the possibility of selective reporting.
